# Implementation of the trial emulation approach in medical research: a scoping review

**DOI:** 10.1186/s12874-023-02000-9

**Published:** 2023-08-16

**Authors:** Giulio Scola, Anca Chis Ster, Daniel Bean, Nilesh Pareek, Richard Emsley, Sabine Landau

**Affiliations:** 1 Department of Biostatistics and Health Informatics, Institute of Psychiatry, Psychology and Neuroscience, King’s College London, London, UK; 2 Health Data Research UK London, Institute of Health Informatics, University College London, London, UK; 3 King’s College Hospital NHS Foundation Trust, London, UK; 4 School of Cardiovascular and Metabolic Medicine & Sciences, BHF Centre of Excellence, King’s College London, London, UK

**Keywords:** Causal inference, Target trial, Trial emulation, Observational data

## Abstract

**Background:**

When conducting randomised controlled trials is impractical, an alternative is to carry out an observational study. However, making valid causal inferences from observational data is challenging because of the risk of several statistical biases. In 2016 Hernán and Robins put forward the ‘target trial framework’ as a guide to best design and analyse observational studies whilst preventing the most common biases. This framework consists of (1) clearly defining a causal question about an intervention, (2) specifying the protocol of the hypothetical trial, and (3) explaining how the observational data will be used to emulate it.

**Methods:**

The aim of this scoping review was to identify and review all explicit attempts of trial emulation studies across all medical fields. Embase, Medline and Web of Science were searched for trial emulation studies published in English from database inception to February 25, 2021. The following information was extracted from studies that were deemed eligible for review: the subject area, the type of observational data that they leveraged, and the statistical methods they used to address the following biases: (A) confounding bias, (B) immortal time bias, and (C) selection bias.

**Results:**

The search resulted in 617 studies, 38 of which we deemed eligible for review. Of those 38 studies, most focused on cardiology, infectious diseases or oncology and the majority used electronic health records/electronic medical records data and cohort studies data. Different statistical methods were used to address confounding at baseline and selection bias, predominantly conditioning on the confounders (*N* = 18/49, 37%) and inverse probability of censoring weighting (*N* = 7/20, 35%) respectively. Different approaches were used to address immortal time bias, assigning individuals to treatment strategies at start of follow-up based on their data available at that specific time (*N* = 21, 55%), using the sequential trial emulations approach (*N* = 11, 29%) or the cloning approach (*N* = 6, 16%).

**Conclusion:**

Different methods can be leveraged to address (A) confounding bias, (B) immortal time bias, and (C) selection bias. When working with observational data, and if possible, the ‘target trial’ framework should be used as it provides a structured conceptual approach to observational research.

**Supplementary Information:**

The online version contains supplementary material available at 10.1186/s12874-023-02000-9.

## Background

In medical research, randomised controlled trials (RCTs) are considered the gold-standard study to evaluate the effectiveness of a treatment [1]. However, RCTs are sometimes not feasible due to factors such as their high cost, and even when viable, can still take too long to provide answers to inform pressing clinical and health policy decisions. In this scenario the careful analysis of observational data might provide an alternative to generate evidence to guide those decisions [2–4].

Observational data is a broad term that includes any patient data, health, and care information collected in non-experimental settings (e.g. RCTs) [5, 6]. In this paper, we make the distinction between two types of observational data: research-generated data and non-research-generated data (Table 1).

**Table 1:** Sources of two different types of observational data

**Research-generated data**	**Non-research generated data**
*Epidemiological studies*	*EHRs/EMRs*
Data from cohort, cross-sectional, and case–control studies.	EHRs are digital records of patients’ medical data. Data stored in EHRs are structured (i.e. tabular data) and unstructured (e.g. free-text in clinical notes or image reports) [7].
*Patient registries*	*National registry*
A patient registry is an organised collection of uniform data to evaluate a pre-specified outcome(s) for a population with a specific disease, condition, or exposure [8].	A national registry collects uniform demographics and/or health related data on all its respective country nationals [9].
*Biobanks*	*Health insurance claims databases*
A biobank collects biological samples and in-depth health information on a specific group of people [10].	A health insurance claims database collects data entered on bills (claims) by hospitals, nursing homes, etc. [11].

In this paper no distinction is made between electronic health records and electronic medical records. This table was adapted from a lecture given by Miguel Hernán [12]

*Abbreviations*: *EHRs* Electronic health records, *EMRs* Electronic medical records

Accurate estimation of treatment effects from observational data is challenging. The main reason for that is the possibility of confounding of the effect of treatment on the clinical outcome(s). Unlike in RCTs, in observational studies, patients are not randomly assigned to treatment groups at baseline. Instead, each patient is prescribed a treatment by a clinician according to their demographic and clinical characteristics (e.g. gender, age, severity of illness etc.), which is likely to result in an unequal distribution of these characteristics across treatment groups. If these characteristics are also prognostic factors for the outcome(s), and hence confounders, they must be accounted for, otherwise this may result in confounding bias [13, 14].

Moreover, poorly designed or ill-thought-out observational studies can result in additional issues due to misalignments in treatment initiation, eligibility, and follow-up periods, as well as loss to follow-up [4, 13, 15]. Bias can result from a misalignment of the start of follow-up, eligibility, and treatment initiation. In a well-designed prospective trial, baseline assessment is carried out just before random allocation to treatment, and participant follow-up starts with randomisation. In contrast, in an observational study of treatment initiation vs. no initiation, there can be a delay between start of follow-up (i.e. when the eligibility criteria are met and the study outcome(s) begin to be considered) and treatment initiation. This will result in a period of follow-up time, commonly referred to as ‘immortal time’, when participants in the treated group specifically cannot have died or experienced the outcome(s) and are essentially ‘immortal’. Participants in the treated group are not truly ‘immortal’ during this period of time; however, they must have survived it (i.e. be alive and event-free) to be initiating treatment [13, 14, 16–19]. Inadequate consideration of this unexposed period of time as part of the design or analysis of the observational study, results in ‘immortal time bias’ [18]. Loss to follow-up in observational studies can lead to selection bias since participants lost to follow-up may systematically differ from those who were not lost to follow-up in terms of their treatment status as well as prognostic variables. If this is not accounted for appropriately in the study’s analysis, it may compromise its validity [3, 20].

Additional complexity arises in observational studies which aim to evaluate the causal effect of a sustained treatment strategy or treatment regimen rather than that of a ‘point treatment’. Treatment regimens often consist of a number of treatments that might be sustained over time, such as repeat prescriptions for human immunodeficiency virus (HIV) [21]. When evaluating the causal effect of a particular treatment regimen, e.g. the causal contrast between continuously being prescribed HIV medication versus no prescription at all, the observed treatment histories may depart from these regimens as clinical decisions to re-prescribe drugs may depend on previous drug responses or side effects. Therefore, in such studies there may be (observable) variables such as intermediate treatment response or side effects that are (i) affected by past treatments, and (ii) drive both future treatments allocations as well as the long-term outcome. Such variables are known as ‘time-varying confounders’ to distinguish them from ‘baseline/pre-treatment confounders’. This statistical issue is often overlooked as more complex analysis methods are needed to avoid bias arising from these confounders [21, 22].

In 2016 Hernán and Robins put forward a solution to avert most of those biases, that is the ‘target trial’ framework. This framework consists of three steps. First, clearly defining a causal question about a treatment. Second, specifying the protocol of the ‘target trial’ (i.e. the eligibility criteria, the treatment strategies being compared (including their start and end times), the assignment procedures, the follow-up period, the outcome(s) of interest, the causal contrast(s) of interest and a plan to estimate them without bias). In other words, the protocol of the RCT you would like to perform but cannot due to impracticality. Last, explaining how the observational data will be used to explicitly emulate it. Meticulously following this structured process step by step when planning observational studies can help prevent biases such as immortal time bias and selection bias. Avoiding confounding bias tends to be more difficult in practice. To emulate randomisation, all baseline (and where relevant time-varying) confounders must be measured. However, there is no guarantee that the observational database contains sufficient information on the confounders. Furthermore, there might be confounders that the study investigator is not aware of and therefore does not attempt to measure nor control for (i.e. unobserved confounders). Hence, successful emulation of randomisation is never guaranteed, and there is no certainty that residual confounding is not present [3]. Nonetheless, the ‘target trial’ framework is a rigorous approach for evaluating treatment effects from observational data.

The aim of this scoping review is to identify and review all explicit attempts of trial emulations across all medical fields. This work will provide an overview of the medical fields that have been covered, the types of observational data that have been most frequently used and the statistical methods that have been employed to address the following biases: (A) confounding bias, (B) immortal time bias, and (C) potential selection bias due loss to follow-up, henceforth simply referred to as selection bias.

## Methods

### Search strategy and selection criteria

Three bibliographic databases (Embase (Ovid), Medline (Ovid) and Web of Science) were searched for studies published in English from database inception (Embase (Ovid): 1974, Medline (Ovid): 1946 and Web of Science: 1900) to February 25, 2021, using predefined search terms. These were related to concepts such as trial emulation and observational data (see file Additional file 1).

The studies’ selection process consisted of two key steps. First, identifying and removing all duplicates. This was done automatically in EndNote X9 [23] and was manually checked and completed by one reviewer (GS). Next, identifying eligible studies based on their titles, abstracts and/or keywords. For a study to be considered eligible, it must explicitly mention in its title, abstract or keywords that it emulated a trial using observational data. One reviewer (GS) systematically checked each study’s title, abstract and keywords.

### Data extraction

One reviewer (GS) extracted the data from the studies. Only when further methodological details were necessary, the studies’ supplementary materials were also checked. A custom Excel spreadsheet was used to record specific information, such as the studies’ subject area, what type of observational data were used, the causal contrast(s) of interest, and the statistical methods used for analysing the primary outcome(s) and for addressing the following biases: (A) confounding bias, (B) immortal time bias and (C) selection bias (see Table 2).

**Table 2:** Data extraction form

Questions	Possible categories
*Subject area*	
What is the study’s subject area?	Cardiology, Oncology, Psychiatry, Neurology, etc.
*Data type*	
Were EHRs or EMRs data used?	Yes or no.
If not, what type of data were used?	Cohort study data, Patient registry data, etc.
Specify the name of the observational database.	Free text.
*Data structure*	
Were structured data used?	Yes or no.
Were unstructured data used?	Yes or no.
If unstructured data were used, were these manually or automatically processed?	Manually or automatically.
*Eligibility criteria*	
What is the target population?	Free text.
*Treatments*	
How many treatments were compared?	Number of treatments.
What treatments were compared?	Free text.
*Outcomes*	
What was(were) the primary outcome(s)?	Free text.
*Follow-up*	
Was the follow-up duration pre-specified?	Yes or no.
*Statistical objectives*	
What is the estimand of interest?	Causal effect of point treatment offer (‘intention-to-treat effect’), causal effect of point treatment receipt (‘per-protocol effect’), causal effect of treatment regimen initiation (‘intention-to-treat effect’) or causal effect of sustained treatment regimen (‘per-protocol effect’).
What was the measurement scale of the outcome(s)?	Continuous, ordinal, binary, time-to-event, other.
Which effect size measure was used to quantify the causal contrast of interest?	Mean difference, odds ratio, hazard ratio, other.
Which statistical method was used for analysing the primary outcome(s)?	Pooled logistic regression, Cox proportional hazards model, etc.
Were sample size or statistical power calculations provided?	Yes or no.
If yes, what was determined?	Power or the effect size.
*Treatment assignment procedures*	
Were treatments administered at one point in time or sustained over time?	Point treatment or treatment regimen.
In either case have pre-initiation confounders been adjusted for?	Yes or no.
If the answer to the last question is ‘yes’, what statistical method has been used for this purpose?	Inclusion of covariates in model, stratification, inverse probability of treatment weighting, propensity score methods, parametric g-formula, other, method not specified.
If treatment regimen, are the investigators interested in the effect of initiating a treatment or the effect of sustaining a treatment?	Initiation or sustained treatment.
If interested in the effect of a sustained treatment, did they account for time-varying confounders?	Yes or no.
If the answer to the last question is ‘yes’, what statistical method has been used for this purpose?	Inverse probability of treatment weighting, parametric g-formula, other, method not specified.
*Other bias handling*	
Was immortal-time bias addressed?	Yes or no.
If yes, how was immortal-time bias handled?	Avoided at the study design stage or using the cloning technique.
Was selection bias due to loss to follow-up addressed explicitly?	Yes or no.
If so, how were missing outcome data handled?	Inverse probability of censoring weighting, multiple imputation, etc.

*Abbreviations*: *EHRs* Electronic Health Records, *EMRs* Electronic Medical Records

### Quality check

A second reviewer (AC) re-screened 100 articles (16%) and extracted data from eight out of the 38 eligible articles (21%) to assess the reliability of study selection and data extraction. There were no disagreements between the first and the second reviewer (GS and AC).

## Results

**Fig. 1:**
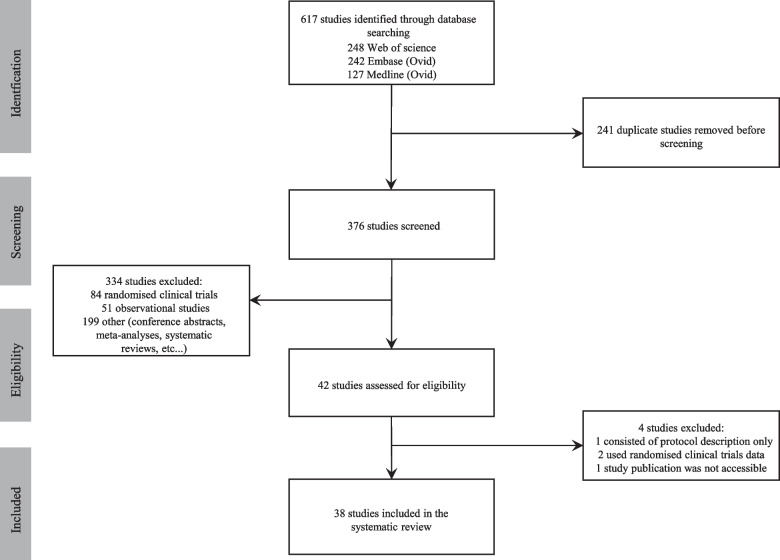
Study selection flow chart

**Fig. 2:**
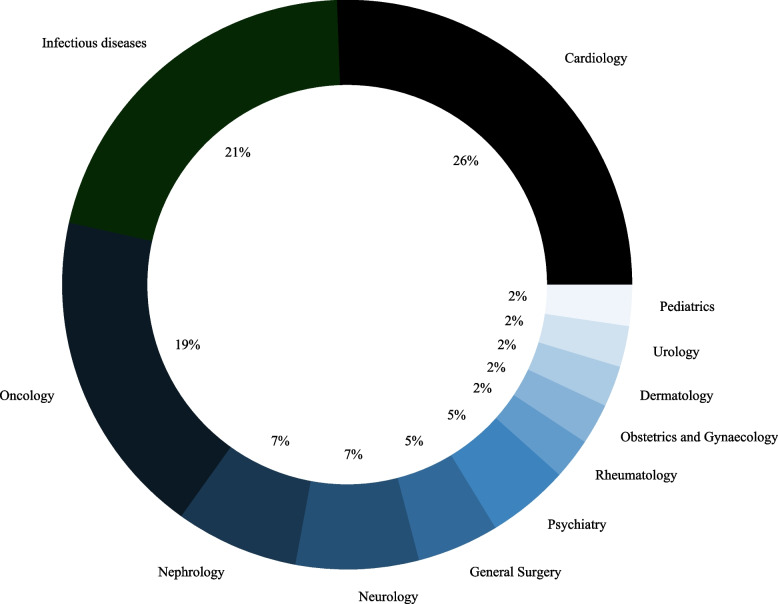
Medical fields most covered *Note.* Studies were classified based on their outcomes, whenever possible

The literature search yielded 617 studies. After removing duplicates and excluding studies based on title, abstract and keywords, 38 studies were identified as eligible for review (Fig. 1). Out of those 38 studies, most were cardiology (*N* = 11, 26%), infectious diseases (*N* = 9, 21%) or oncology (*N* = 8, 19%) studies (Fig. 2). Five studies (9, 23, 31, 35 and 36 in Table 3) covered more than one medical field, and therefore the percentages were calculated out of 43 datasets rather than 38.

**Table 3:** Types of observational data used and subject area

Index	Study	Study’s subject area	Data	Description	Category
1	Dickerman et al. [24]	Oncology	CALIBER	The CALIBER platform (https://www.caliberresearch.org/portal) consists of ‘research ready’ variables extracted from specific structured UK EHRs data sources: primary care (CPRD), hospitalizations (HES) and mortality (ONS).	EHRs/EMRs data
2	García-Albéniz et al. [25]	Oncology	(SEER)-Medicare linked database	The SEER-Medicare database (https://healthcaredelivery.cancer.gov/seermedicare/) consists of cancer patients’ data collected by 17 SEER cancer registries across 12 US states as well as Medicare claims data collected by the Centres for Medicare & Medicaid Services.	1. Health insurance claims data 2. Patient registry data
3	Petito et al. [26]	Oncology	(SEER)-Medicare linked database	Explained previously (see row 2).	1. Health insurance claims data 2. Patient registry data
4	Dickerman et al. [4]	Oncology	CALIBER	Explained previously (see row 1).	EHRs/EMRs data
5	Dickerman et al. [27]	Oncology	HPFS	The HPFS (https://sites.sph.harvard.edu/hpfs/) is an ongoing prospective cohort study of over 50,000 US male health professionals aged between 40–75 years at enrolment in 1986.	Cohort study data
6	Danaei et al. [28]	Cardiology	THIN	The THIN database (https://www.the-health-improvement-network.com/) consists of EHRs data from over 500 primary care practices in the UK.	EHRs/EMRs data
7	Zhang et al. [29]	Cardiology	USRDS	The USRDS (https://www.usrds.org/) is a national data system that collects data on US patients with disease CKD and ESRD.	Patient registry data
8	Atkinson et al. [30]	Infectious diseases	COHERE	COHERE (www.cohere.org) is a collaboration of 40 HIV European cohort studies.	Cohort study data
9	Rojas‑Saunero et al. [31]	1. Neurology 2. Cardiology	The Rotterdam study	The Rotterdam study (https://www.ergo-onderzoek.nl/) is an ongoing prospective cohort study that started in 1990 in Ommoord, a suburb of Rotterdam, the Netherlands. As of 2008, the cohort consists of approximately 15,000 subjects aged 45 years and over.	Cohort study data
10	Maringe et al. [14]	Oncology	NCRAS and secondary administrative records	The NCRAS (https://www.gov.uk/guidance/national-cancer-registration-and-analysis-service-ncras) collects data on cancer patients living in England.	Patient registry data
11	Gilbert et al. [32]	Psychiatry	Neptune	The Neptune (https://www.dbmi.pitt.edu/services/) system consists of EMRs data.	EHRs/EMRs data
12	Caniglia et al. [33]	Psychiatry	VACS	The VACS (https://www.vacsp.research.va.gov/CSPEC/Studies/INVESTD-R/Veteran-Aging-Cohort-Study.asp) is an ongoing prospective cohort study of HIV-positive and age/race/site matched control group of HIV-negative US veterans in care, launched in 1997.	Cohort study data
13	Althunian et al. [34]	Cardiology	CPRD	The CPRD database (https://cprd.com/) consists of EHRs data collected from a UK-wide network of primary care practices.	EHRs/EMRs data
14	Shaefi et al. [35]	Infectious disease	STOP-COVID	STOP-COVID (https://clinicaltrials.gov/ct2/show/NCT04343898) is a multicentre cohort study of COVID-19 patients (≥ 18 years old) admitted to participating intensive care units across the US.	Cohort study data
15	Bacic et al. [36]	Oncology	NCDB	The NCDB ( https://www.facs.org/quality-programs/cancer-programs/national-cancer-database/) is a clinical oncology database that collects cancer patients’ hospital registry data from over 1,500 hospitals in the US.	Patient registry data
16	Rossides et al. [37]	Infectious diseases	Swedish register data (PDR, NPR, and other registers)	The Swedish PDR https://www.socialstyrelsen.se/en/statistics-and-data/registers/national-prescribed-drug-register/) contains information about all drug prescriptions dispensed in Sweden since July 2005. The Swedish NPR (https://www.socialstyrelsen.se/en/statistics-and-data/registers/national-patient-register/) contains information on in-patients at public hospitals.	National registry data
17	Xie et al. [38]	Nephrology	VA databases	VA databases collect data on US veterans who are enrolled in the VA health care system (https://www.va.gov/health-care/).	EHRs/EMRs data
18	Caniglia et al. [39]	Neurology	The Rotterdam study	Explained previously (see row 9)	Cohort study data
19	Caniglia et al. [40]	Infectious diseases	HIV -CAUSAL	HIV-CAUSAL (https://causalab.sph.harvard.edu/hiv/) is a collaboration of European and American HIV prospective cohort studies	Cohort study data
20	Caniglia et al. [41]	Gynaecology and obstetrics	Used data from a birth surveillance study in Botswana	-	Cohort study data
21	Matthews et al. [42]	Infectious diseases	STOP-COVID	Explained previously (see row14).	Cohort study data
22	Schmidt et al. [43]	Cardiology	Danish National Patient & Prescription Registers	The Danish National Patient Register contains data on people who have been admitted to somatic (since 1977) ambulatory and emergency (since 1995) hospital departments. The Danish National Prescriptions Registry contains information on all prescription drugs sold in Danish community pharmacies since 1994 [43].	National registry data
23	Al-Samkari et al. [44]	1. Cardiology 2. Infectious diseases	STOP-COVID	Explained previously (see row 14).	Cohort study data
24	Mattishent et al. [45]	Cardiology	CPRD	Explained previously (see row 13).	EHRs/EMRs data
25	Lenain et al. [46]	General surgery	REIN registry	The REIN registry ( https://clinicaltrials.gov/ct2/show/NCT03967808) was set up in 2002. It collects data on ESRD patients on replacement therapy – either dialysis or transplantation -living in metropolitan France or in overseas districts.	Patient registry data
26	Yiu et al. [47]	Dermatology	BADBIR	The BADBIR database (http://www.badbir.org/) consists of psoriasis patients’ data undergoing treatment with either a biologic drug or a standard anti-psoriatic therapy in the UK and the ROI.	Patient registry data
27	Wanis et al. [48]	General surgery	SRTR	The SRTR database (https://www.srtr.org/) consists of organ donors, transplant recipients and organ transplant wait-listed candidates’ data in the US, submitted by the Organ Procurement and Transplant Network.	Patient registry data
28	Lu et al. [49]	Infectious disease	NA-ACCORD	The NA-ACCORD study ( https://naaccord.org/) is a collaboration of North American HIV prospective cohort studies.	Cohort study data
29	Lyu et al. [50]	Rheumatology	THIN	Explained previously (see row 6).	EHRs/EMRs data
30	Russell et al. [51]	Oncology	BladderBaSe	BladderBaSe was set up back in 2015. It links information from the SNRUBC with several national healthcare and demographic register [51].	National registry data
31	Takeuchi et al. [52]	1. Urology 2. Infectious diseases	NDB	NDB collects claims data from almost all Japanese citizens and long-term residents of Japan [52].	Health insurance claims data
32	Abrahami et al. [53]	Cardiology	CPRD and ONS	Explained previously (see rows 1 and 13).	EHRs/EMRs data
33	Secora et al. [54]	Nephrology	GHS	GHS ( https://www.geisinger.org/) is a healthcare provider in Pennsylvania. Its EHR database contains information on more than 3 million patients.	EHRs/EMRs data
34	Young et al. [55]	Infectious diseases	SHCS	The SHCS ( http://www.shcs.ch/) is an ongoing prospective cohort study of HIV-positive patients (≥ 16 years old) that was launched in 1988.	Cohort study data
35	Czaja et al. [56]	1. Cardiology 2. Pediatrics	CER^2^	CER^2^ (https://dartnet.info/CER2.htm) network has collected EHRs data from over 1 million paediatric patients across 27 states in the US.	EHRs/EMRs data
36	Keyhani et al. [57]	1. Cardiology 2. Neurology	Medicare and VA databases	Medicare (https://www.medicare.gov/) is a US federal health insurance scheme that subsidises healthcare services for US citizens aged 65 years or over. VA databases was explained previously (see row 17)	1. Health insurance claims data 2. EHRs and EMRs data
37	Franklin et al. [58]	Cardiology	3 US health care claims data sources: Optum Clinformatics, IBM MarketScan and Medicare	Optum Clinformatics and IBM MarketScan are two commercial US claims database [58].	Health insurance claims data
38	Fu et al. [59]	Nephrology	The Swedish Renal Registry	The Swedish Renal Registry is a nationwide registry of patients with stages 3–5 CKD who have attended nephrologist-specialist care in Sweden between 2007–2017 [59].	National registry data

*Abbreviations*: *CALIBER* ClinicAI research using Linked Bespoke studies and Electronic health Records, *CPRD* Clinical Practice Research Datalink, *HES* Hospital Episode Statistics, *ONS* Office for National Statistics, *EHRs* electronic health records, *EMRs* electronic medical Records, *SEER* Surveillance, Epidemiology and End Results program, *US* United States, *HPFS* Health Professionals Follow-up Study, *THIN* The Health Improvement Network, *UK* United Kingdom, *USRDS* United States Renal Data System, *CKD* chronic kidney disease, *ESRD* end-stage renal disease, *COHERE* the Collaboration of Observational HIV Epidemiological Research in Europe, *HIV* Human immunodeficiency virus, *NCRAS* National Cancer Registration and Analysis Service, *VACS* the Veterans Aging Cohort Study, *STOP-COVID* The Study of the Treatment and Outcomes in Critically Ill Patients with COVID-19, *COVID-19* coronavirus disease, *ICUs* intensive care units, *NCDB* National Cancer Database, *PDR* Prescribed Drug Register, *NPR* National Patient Register, *VA* Department of Veterans Affairs, *HIV-CAUSAL* HIV Cohorts Analyzed Using Structural Approaches to Longitudinal data, *REIN* the French Renal Epidemiology and Information Network, *RRT* renal replacement therapies, *BADBIR* British Association of Dermatologists Biologic and Immunomodulators Register, *ROI* Republic of Ireland, *SRTR* Scientific Registry of Transplant Recipients, *OPTN* Organ Procurement and Transplant Network, *NA-ACCORD* the North American AIDS Cohort Collaboration on Research and Design, *BladderBaSe* the Bladder Cancer Data Base Sweden, *SNRUBC* The Swedish National Register of Urinary Bladder Cancer, *NDB* the National Database of Health Insurance Claims and Specific Health Check-ups of Japan, *GHS* Geisinger Health System, *SHCS* Swiss HIV Cohort Study, *CER*^*2*^ The Comparative Effectiveness Research through Collaborative Electronic Reporting Consortium

### Observational data sources

Out of the 38 studies we reviewed, most used electronic health records (EHRs)/electronic medical records (EMRs) data (*N* = 12, 29%) and cohort studies data (*N* = 12, 29%) (see Table 3). Among those that used EHRs/EMRs data, only Keyhani and colleagues mentioned using a natural language processing (NLP) algorithm to retrieve and extract unstructured data, i.e. ‘carotid imaging results showing stenosis of less than 50% or hemodynamically insignificant stenosis’ [57]. Three studies (2, 3 and 36 in Table 3) used different observational data sources, and therefore the percentages were calculated out of 41 datasets rather than 38.

### Causal contrast of interest

Most of the trial emulation studies we reviewed aimed to assess the causal effect of treatment initiation – the observational analogue of the intention-to-treat effect (ITT) in trials (25 out of 38 studies reviewed, with 21 out of those 25 considering the initiation of a treatment regimen rather than point treatments). Seven studies assessed the causal effect of receiving a point treatment and 15 studies compared the effect of two or more alternative sustained treatment regimens including no treatment—the observational analogue of a per-protocol (PP) effect. Nine studies (1, 4, 6, 13, 17, 18, 26, 28 and 31 in Table 4) assessed both types of causal contrasts.

**Table 4:** Causal contrast of interest and methods used to address different biases

Index	Study	The estimand of interest	The measurement scale of the outcome(s)	The effect size measure used to quantify the causal contrast of interest	The statistical method used for analysing the primary outcome(s)	The statistical method used to adjust for baseline confounders	The statistical method used to account for time-varying confounders	The approach used to address immortal-time bias	The statistical method used to account for potential selection bias due to loss to follow-up
1a (cohort analysis)	Dickerman et al. [24]	ITT PP (treatment regimen)	Time-to-event	HR RD	Pooled logistic regression	Outcome regression on the confounders	IPTW	Participants assigned to treatment groups at start of follow-up based on their data available at that time	IPCW
1b (case–control analysis)	Dickerman et al. [24]	ITT PP (treatment regimen)	Time-to-event	OR	Pooled logistic regression	Outcome regression on the confounders	IPTW	Cases and controls were sampled from the assembled cohort	IPCW
2	García-Albéniz et al. [25]	ITT (point treatment)	Time-to-event	RD	Pooled logistic regression	Outcome regression on the confounders	N.A.	Sequential trial emulations approach	Could not be determined
3a (the addition of fluorouracil in stage II colorectal cancer)	Petito et al. [26]	PP (point treatment)	Time-to-event	HR RD	Pooled logistic regression	1. Cloning approach + IPCW 2. Outcome regression on the confounders	N.A.	Cloning approach + IPCW	Could not be determined
3b (the use of erlotinib in advanced pancreatic adenocarcinoma)	Petito et al. [26]	PP (point treatment)	Time-to-event	HR RD	Pooled logistic regression	1. Cloning approach + IPCW 2. Outcome regression on the confounders	N.A.	Cloning approach + IPCW	Could not be determined
4	Dickerman et al. [4]	ITT PP (treatment regimen)	Time-to-event	HR SD	Pooled logistic regression	Outcome regression on the confounders	IPTW	Sequential trial emulations approach	IPCW
5	Dickerman et al. [27]	PP (treatment regimen)	Time-to-event	RR RD	PGF	PGF	PGF	Participants assigned to treatment groups at start of follow-up based on their data available at that time	PGF
6a (single treatment versus no treatment)	Danaei et al. [28]	ITT PP (treatment regimen)	Time-to-event	HR SD	Pooled logistic regression	Outcome regression on the confounders	IPTW	Sequential trial emulations approach	Could not be determined
6b (joint treatment versus no treatment)	Danaei et al. [28]	ITT PP (treatment regimen)	Time-to-event	HR SD	Pooled logistic regression	Outcome regression on the confounders	IPTW	Sequential trial emulations approach	Could not be determined
6c (head-to-head comparison of two treatments)	Danaei et al. [28]	ITT PP (treatment regimen)	Time-to-event	HR SD	Pooled logistic regression	Outcome regression on the confounders	IPTW	Sequential trial emulations approach	Could not be determined
7	Zhang et al. [29]	PP (treatment regimen)	Time-to-event	RR RD	PGF	PGF	PGF	Participants assigned to treatment groups at start of follow-up based on their data available at that time	PGF
8	Atkinson et al. [30]	PP (point treatment)	Time-to-event	HR	Pooled logistic regression	1. Cloning approach + IPCW 2. Outcome regression on the confounders	N.A.	Cloning approach + IPCW	Could not be determined
9	Rojas‑Saunero et al. [31]	PP (treatment regimen)	Time-to-event	RR RD	PGF	PGF	PGF	Participants assigned to treatment groups at start of follow-up based on their data available at that time	PGF
10	Maringe et al. [14]	PP (point treatment)	Time-to-event	SD	Kaplan–Meier estimator	Cloning approach + IPCW	N.A.	Cloning approach + IPCW	CCA
11	Gilbert et al. [32]	PP (treatment regimen)	Time-to-event	HR	Pooled logistic regression	Outcome regression on the confounders	IPTW	Participants assigned to treatment groups at start of follow-up based on their data available at that time	Could not be determined
12	Caniglia et al. [33]	PP^a^ (point treatment)	Binary	OR	Logistic regression	IPTW	N.A.	Participants assigned to treatment groups at start of follow-up based on their data available at that time	Could not be determined
13	Althunian et al. [34]	ITT PP (treatment regimen)	Time-to-event	HR	Cox proportional hazards model	Outcome regression on the confounders	Could not be determined	Participants assigned to treatment groups at start of follow-up based on their data available at that time	Could not be determined
14	Shaefi et al. [35]	ITT^a^ (treatment regimen)	Time-to-event	HR	Cox proportional hazards model	Outcome regression on the confounders	N.A.	Sequential trial emulations approach	Could not be determined
15a (index trial emulation)	Bacic et al. [36]	ITT^a^ (point treatment)	Time-to-event	HR	Cox proportional hazards model	IPTW	N.A.	Participants assigned to treatment groups at start of follow-up based on their data available at that time	Could not be determined
15b (high risk trial emulation)	Bacic et al. [36]	ITT^a^ (point treatment)	Time-to-event	HR	Cox proportional hazards model	IPTW	N.A.	Participants assigned to treatment groups at start of follow-up based on their data available at that time	Could not be determined
16	Rossides et al. [37]	ITT (treatment regimen)	Binary	RR RD	TMLE	TMLE	N.A.	Sequential trial emulations approach	TMLE
17	Xie et al. [38]	ITT PP (treatment regimen)	Time-to-event	HR	1. Cox proportional hazards model (ITT) 2. Pooled logistic regression (PP)	1. GPS (ITT) 2. IPTW (PP)	IPTW	Participants assigned to treatment groups at start of follow-up based on their data available at that time	Could not be determined
18	Caniglia et al. [39]	ITT PP (treatment regimen)	Time-to-event	RD	Pooled logistic regression	Outcome regression on the confounders	IPTW	Sequential trial emulations approach	IPCW
19	Caniglia et al. [40]	PP (treatment regimen)	Time-to-event	SD	Pooled logistic regression	1. Cloning approach + IPCW 2. Outcome regression on the confounders	Cloning approach + IPCW	Cloning approach + IPCW	Could not be determined
20a (historical comparison)	Caniglia et al. [41]	Modified ITT (treatment regimen)	Binary	RR	1. Log-binomial regression 2. Poisson regression	1. Adjusted for confounders at the design stage 2. Outcome regression on the confounders	N.A.	Participants assigned to treatment groups at start of follow-up based on their data available at that time	IPCW
20b (﻿contemporaneous comparison)	Caniglia et al. [41]	Modified ITT (treatment regimen)	Binary	RR	1. Log-binomial regression 2. Poisson regression	1. Adjusted for confounders at the design stage 2. Outcome regression on the confounders	N.A.	Participants assigned to treatment groups at start of follow-up based on their data available at that time	IPCW
21	Matthews et al. [42]	ITT^a^ (treatment regimen)	Time-to-event	HR	Cox proportional hazards model	IPTW	N.A.	Sequential trial emulations approach	Could not be determined
22	Schmidt et al. [43]	ITT (treatment regimen)	Time-to-event	HR	Cox proportional hazards model	1. Propensity score matching 2. Outcome regression on the confounders	N.A.	Sequential trial emulations approach	CCA
23	Al-Samkari et al. [44]	ITT (treatment regimen)	Time-to-event	HR	Cox proportional hazards model	IPTW	N.A.	Sequential trial emulations approach	Could not be determined
24a (test the effect of hypoglycemia among individuals with dementia and diabetes, with respect to subsequent serious adverse events)	Mattishent et al. [45]	PP^a^ (point treatment)	Time-to-event	HR	Cox proportional hazards model	Outcome regression on the confounders	N.A.	Participants assigned to treatment groups at start of follow-up based on their data available at that time	1. CCA 2. MI
24b (evaluate whether the effect of hypoglycemia was modified by the presence or absence of dementia)	Mattishent et al. [45]	PP^a^ (point treatment)	Time-to-event	HR	Cox proportional hazards model	Outcome regression on the confounders	N.A.	Participants assigned to treatment groups at start of follow-up based on their data available at that time	1. CCA 2. MI
25	Lenain et al. [46]	ITT (point treatment)	Time-to-event	SD	Kaplan–Meier estimator	Matching on time-dependent propensity score	N.A.	Participants assigned to treatment groups at start of follow-up based on their data available at that time	CCA
26	Yiu et al. [47]	ITT PP (treatment regimen)	Binary	RD RR	Generalized linear model	1. Propensity score matching 2. IPTW	IPTW	Participants assigned to treatment groups at start of follow-up based on their data available at that time	1. CCA 2. Nonresponder imputation 3. Last observation carried forward 4. IPCW 5. MI
27	Wanis et al. [48]	ITT (point treatment)	Time-to-evet	SD	1. Kaplan–Meier estimator 2. Pooled logistic regression	Outcome regression on the confounders (pooled logistic regression)	N.A.	Participants assigned to treatment groups at start of follow-up based on their data available at that time	Could not be determined
28	Lu et al. [49]	ITT PP (treatment regimen)	Time-to-event	HR RD	1. Cox proportional hazards model 2. Weighted Kaplan–Meier estimator	IPTW	IPTW	Participants assigned to treatment groups at start of follow-up based on their data available at that time	IPCW
29	Lyu et al. [50]	PP (point treatment)	Time-to-event	HR RD	Pooled logistic regression	1. Cloning approach + IPCW 2. Outcome regression on the confounders	N.A.	Cloning approach + IPCW	IPCW
30	Russell et al. [51]	ITT (treatment regimen)	Time-to-event	HR	Cox proportional hazards model	1. Propensity score matching 2. Outcome regression on the confounders	N.A.	Participants assigned to treatment groups at start of follow-up based on their data available at that time	Could not be determined
31	Takeuchi et al. [52]	ITT PP (treatment regimen)	Time-to-event	HR	Cox proportional hazards model	IPTW	IPTW	Participants assigned to treatment groups at start of follow-up based on their data available at that time	Could not be determined
32	Abrahami et al. [53]	ITT (treatment regimen)	Time-to-event	HR	Cox proportional hazards model	Propensity score methods (adjustment, stratification, fine stratification and matching)	N.A.	Participants assigned to treatment groups at start of follow-up based on their data available at that time	Could not be determined
33	Secora et al. [54]	ITT (treatment regimen)	Time-to-event	HR	Time-to-event Fine and Gray regression model	1. Outcome regression on the confounders 2. IPTW 3. Propensity score matching	N.A.	Sequential trial emulations approach	Could not be determined
34a (comparison of partly NRTI-sparing regimens)	Young et al. [55]	ITT^a^ (treatment regimen)	Time-to-event	HR	Bayesian Cox proportional hazards model	Propensity score matching	N.A.	Participants assigned to treatment groups at start of follow-up based on their data available at that time	Could not be determined
34b (comparison of fully NRTI-sparing regimens)	Young et al. [55]	ITT^a^ (treatment regimen)	Time-to-event	HR	Bayesian Cox proportional hazards model	Propensity score matching	N.A.	Participants assigned to treatment groups at start of follow-up based on their data available at that time	Could not be determined
35	Czaja et al. [56]	ITT^a^ (treatment regimen)	Time-to-event	OR	Pooled logistic regression	IPTW	N.A.	Sequential trial emulations approach	Could not be determined
36	Keyhani et al. [57]	PP^a^ (point treatment)	Time-to-event	RD	Kaplan–Meier estimator	Propensity score matching	N.A.	Participants assigned to treatment groups at start of follow-up based on their data available at that time	Could not be determined
37a (LEADER)	Franklin et al. [58]	ITT (treatment regimen)	Time-to-event	HR	Cox proportional hazards model	Propensity score matching	N.A.	Participants assigned to treatment groups at start of follow-up based on their data available at that time	Could not be determined
37b (DECLARE)	Franklin et al. [58]	ITT (treatment regimen)	Time-to-event	HR	Cox proportional hazards model	Propensity score matching	N.A.	Participants assigned to treatment groups at start of follow-up based on their data available at that time	Could not be determined
37c (EMPA-REG)	Franklin et al. [58]	ITT (treatment regimen)	Time-to-event	HR	Cox proportional hazards model	Propensity score matching	N.A.	Participants assigned to treatment groups at start of follow-up based on their data available at that time	Could not be determined
37d (CANVAS)	Franklin et al. [58]	ITT (treatment regimen)	Time-to-event	HR	Cox proportional hazards model	Propensity score matching	N.A.	Participants assigned to treatment groups at start of follow-up based on their data available at that time	Could not be determined
37e (CARMELINA)	Franklin et al. [58]	ITT (treatment regimen)	Time-to-event	HR	Cox proportional hazards model	Propensity score matching	N.A.	Participants assigned to treatment groups at start of follow-up based on their data available at that time	Could not be determined
37f (TECOS)	Franklin et al. [58]	ITT (treatment regimen)	Time-to-event	HR	Cox proportional hazards model	Propensity score matching	N.A.	Participants assigned to treatment groups at start of follow-up based on their data available at that time	Could not be determined
37 g (SAVOR- TIMI)	Franklin et al. [58]	ITT (treatment regimen)	Time-to-event	HR	Cox proportional hazards model	Propensity score matching	N.A.	Participants assigned to treatment groups at start of follow-up based on their data available at that time	Could not be determined
37 h (CAROLINA)	Franklin et al. [58]	ITT (treatment regimen)	Time-to-event	HR	Cox proportional hazards model	Propensity score matching	N.A.	Participants assigned to treatment groups at start of follow-up based on their data available at that time	Could not be determined
37i (TRITON- TIMI)	Franklin et al. [58]	ITT (treatment regimen)	Time-to-event	HR	Cox proportional hazards model	Propensity score matching	N.A.	Participants assigned to treatment groups at start of follow-up based on their data available at that time	Could not be determined
37j (PLATO)	Franklin et al. [58]	ITT (treatment regimen)	Time-to-event	HR	Cox proportional hazards model	Propensity score matching	N.A.	Participants assigned to treatment groups at start of follow-up based on their data available at that time	Could not be determined
38	Fu et al. [59]	PP (treatment regimen)	Time-to-event	RD	Pooled logistic regression	Cloning approach + IPCW	Cloning approach + IPCW	Cloning approach + IPCW	Could not be determined

*Abbreviations*: *ITT* Intention-to-treat effect, *PP* Per-protocol effect, *HR* Hazard ratio, *RD* Risk difference, *IPTW* Inverse probability of treatment weighting, *IPWC* Inverse probability of censoring weighting, *OR* Odds ratio, *SD* Survival difference, *RR* Risk ratio, *PGF* Parametric g-formula, *CCA* Complete case analysis, *TMLE* Targeted maximum likelihood estimation, *GPS* Generalised propensity scores, *MI* Multiple imputation

The symbol ‘^a^’ indicates that the information is not explicitly stated and was assumed given the methodological details provided

Most of the primary outcomes of the reviewed studies were measured on a time-to-event scale (*N* = 34/38, 89%). As a result, the most common effect size measure used was the hazard ratio (*N* = 22, 65%), which was estimated by fitting a Cox proportional hazards model (*N* = 14, 61%), a pooled logistic regression (*N* = 8, 35%) or a time-to-event Fine and Gray regression model (*N* = 1, 4%). One study used both a Cox proportional hazards model and a pooled logistic regression, which resulted in the calculation of percentages based on 23 datasets instead of 22 (17 in Table 4).

### Handling of confounding

When estimating the observational analogue of an ITT effect, trial emulation studies used different statistical methods to adjust for baseline confounders, such as conditioning on the confounders (*N* = 18, 37%), propensity score methods (propensity score matching, stratification on the propensity score and adjustment based on the propensity score, etc., *N* = 10, 20%), and g-methods: inverse probability of treatment weighting (IPTW, *N* = 10, 20%), the parametric g-formula (*N* = 3, 6%) and doubly robust methods, i.e. targeted maximum likelihood estimation (TMLE, *N* = 1, 2%). Six studies (12%) used the cloning approach in combination with inverse probability of censoring weighting (IPCW), as suggested by Hernán within the context of the ‘target trial’ framework (3, 8, 10, 19, 29 and 38 in Table 4). Out of these six studies, four additionally conditioned on confounders in their analyses (3, 8, 19 and 29 in Table 4). Despite trying to adjust for confounders at the design stage, one study (2%) still relied on conditioning on those confounders in their analyses (20 in Table 4). Ten studies used more than one method, and therefore the percentages were calculated out of 49 datasets rather than 38 (3, 8, 17, 19, 20, 22, 26, 29, 30, and 33 in Table 4).

Out of the 15 studies that reported the observational analogue of the PP effect for sustained treatment strategies most used g-methods to adjust for time varying-confounding. More specifically, nine studies (60%) used IPTW, two studies (13%) used the cloning approach combined with IPCW, and an additional three studies (20%) used the parametric g-formula. For one study (7%) it was unclear which statistical method they had used (13 in Table 4).

### Immortal time bias

All studies reviewed attempted to address immortal time bias. This was achieved in on one of three ways: (1) by designing studies so that participants are assigned to treatment strategies at start of follow-up based on their data available at that specific time (*N* = 21, 55%), (2) using the cloning approach (*N* = 6, 16%) or (3) by using the sequential trial emulations approach (*N* = 11, 29%) (Table 4).

### Selection bias

Out of the 38 reviewed studies, only 15 studies (39%) explicitly addressed the possibility of selection bias resulting from loss to follow-up. These studies used different methods including IPCW (*N* = 7, 35%), the parametric g-formula (*N* = 3, 15%), TMLE (*N* = 1, 5%), multiple imputation (*N* = 2, 10%), last observation carried forward (*N* = 1, 5%), non-responder imputation (*N* = 1, 5%), and a complete case analysis (*N* = 5, 25%). Two studies used multiple methods, and therefore the percentages were calculated out of 20 datasets rather than 15 (24 and 26 in Table 4). For the remaining 25 studies (61%) it was unclear whether and how they adjusted for selection bias (see Table 4).

## Discussion

Out of the 38 trial emulation studies we reviewed, most concerned cardiology, infectious diseases, and oncology. Furthermore, those studies leveraged different types of observational data, predominantly EHRs/EMRs data and cohort study data. It is worth noting that among those studies that used EHRs/EMRs data, only one study mentioned using unstructured EHRs/EMRs data. However, we do not exclude the possibility of some EHRs/EMRs databases having already pre-processed and converted unstructured EHRs/EMRs data to a structured tabular format.

The reviewed trial emulation studies used conventional or more advanced statistical methods to adjust for baseline confounders when estimating the observational analog of an ITT effect. Conventional statistical methods include conditioning on the putative confounders (i.e. including the confounding variables in the statistical model), whereas more advanced statistical methods include propensity score methods and g-methods (IPTW, the parametric g-formula and TMLE).

Conversely, when estimating the observational analog of the PP effect of sustained treatment strategies, the reviewed studies used g-methods, specifically IPTW and the parametric g-formula, to account for time-varying confounders. Such more advanced statistical methods were needed because time-varying confounders can themselves be affected by prior treatment and adjusting for them using conventional statistical or propensity score methods would prevent the identification of the total causal effect of treatment.

In summary, both conventional and more advanced statistical methods can be used to adjust for confounding at baseline. However, to properly account for time-varying confounding, specific statistical methods, such as the parametric g-formula and IPTW must be used.

To address immortal time bias different approaches can be used. One common approach is to assign individuals to treatment strategies at the start of follow-up based on their data available at that specific time. Additionally, alternative approaches, such as the sequential trial emulation approach or the cloning approach, can be used.

Start of follow-up is the time when an individual meets the eligibility criteria and is assigned a treatment strategy. In some instances, however, an individual might meet the eligibility criteria at multiple times. For example, when comparing initiators and non-initiators of treatment, a non-initiator at one specific point in time might be an initiator at a subsequent point in time and meet the eligibility criteria at both time points. When that is the case, there are two unbiased options for choosing the start of follow-up. One option is to consider a single eligible time point. The other is to consider both time points and use the sequential trial emulation approach. This consists in emulating a sequence of trials, with different starts of follow-up, thereby making it possible for a non-initiator to enter a subsequent trial as an initiator if they meet all the eligibility criteria at the start of that subsequent trial. It should be noted, however, that since the same individuals might contribute to multiple emulated trials, the variance estimators must be adjusted for appropriately. Furthermore, emulating a sequence of trials is expected to yield more precise results compared to emulating a single trial, given the additional data available for analysis [3, 60].

As regards the cloning approach, it is used when the treatment strategies of the individuals are unknown at baseline. It consists of three key steps for implementation. First, in the case of a trial emulation study with two treatment groups under study, if individuals cannot yet be assigned to a specific treatment strategy at baseline, two exact copies (clones) of each individual are created. One clone is assigned to one treatment group, whilst the other is assigned to the other treatment group. Next, clones are followed over time and are censored when they deviate from their assigned treatment strategy. Last, IPCW is used to account for potential selection bias resulting from censoring [14, 60]. Given that only clones who comply with their assigned treatment strategy are kept under study, the cloning approach only allows for the estimation of the observational analog of the PP effect in trial emulations with point treatments or sustained treatment strategies. Furthermore, the cloning approach can be used in combination with a grace period. This is a predefined time period of the follow-up during which treatment initiation can happen and its length is chosen based on real-world clinical scenarios (e.g. hospital delays before surgery). Using the grace period makes it possible to better reflect real-world clinical scenarios and can increase the number of eligible individuals from the observational database [3, 14, 61]. In relation to confounding bias when using the cloning approach, cloning patients removes confounding at baseline. However, artificially censoring clones introduces selection bias, which is accounted for using IPCW [14, 60]. Nonetheless, most of the studies using the cloning approach still adjusted for confounders at baseline.

In summary, different strategies can be used to address immortal time bias, assigning individuals to treatment strategies at baseline based on their data available at that specific time; using the sequential trial emulations approach or the cloning approach.

Potential selection bias resulting from loss to follow-up was primarily accounted for using IPCW. Other methods include complete case analysis, the parametric g-formula, TMLE, multiple imputation, last observation carried forward, and non-responder imputation.

As a general remark, it should be noted that not all trial emulation studies we reviewed have mentioned explicitly using the ‘target trial’ framework, or if they did use it, have not reported the use of it clearly. Those that did use the ‘target trial’ framework tended to follow its reporting guidelines, usually provided a table in their papers outlining the protocol of the ‘target trial’ and explicitly specifying how each component of its protocol was emulated using observational data. Reporting these details is crucial, and is advised going forward, as it allows readers to readily understand the aim of the study and the statistical methods used to address confounding bias, immortal time bias and selection bias.

### Limitations

This scoping review has one main limitation which is that our search strategy has most certainly not identified all trial emulation studies published by February 25, 2021. This is a result of varying nomenclature – where not every trial emulation study refers to itself as such. For instance, to our knowledge, the first ever trial emulation study that was published was defined as an: ‘observational study analysed like a randomised experiment’ [2]. We refrained from using search terms like ‘randomised experiment’ and/or ‘randomised clinical trial’ in our search strategy because, when combined with search terms such as ‘observational study’ and/or ‘observational data’, our search strategy would yield thousands of studies, which for the most part would be most likely irrelevant. Instead, we decided to use search terms such as ‘trial emulation’ and ‘target trial’, which were coined by Hernán and Robins in 2016, who were the first to formalise the idea of using observational data to emulate a randomised trial. This, however, could have resulted in omitting some trial emulation studies, as we acknowledge the fact that not every researcher/research group might refer to trial emulation as such. Future trial emulations work should clearly label themselves as such going forward, both in their abstracts and throughout their papers.

### Future directions

Currently there is much interest regarding the suitability of EHRs/EMRs data for trial emulation purposes given the increased availability of big electronic healthcare databases. The main concern is the quality of EHRs/EMRs data. These should be free from errors, inconsistencies and inaccuracies, and provide all the information required to answer the causal research question under study, including data on exposure, outcome, baseline confounders, time-varying confounders (if applicable), eligibility criteria and missingness predictors. Furthermore, the data should be available in standardized format, trustworthy, and up-to-date [3, 4, 62].

Trial emulation studies that have used EHRs/EMRs data, extracted data from multiple sources. For instance, The Health Improvement Network database, which was used in some studies, consists of EHRs/EMRs data from over 500 primary care practices in the United Kingdom (UK) [63]. This type of EHR/EMR database has proved useful for research purposes. It remains to be determined, however, whether EHRs/EMRs data from a single healthcare facility can be used successfully to emulate trials, inform clinical decisions, and ultimately contribute to improving patient care at the facility itself. In England specifically, large National Health Service (NHS) Trusts, such as King’s College Hospital, the University College London Hospitals, and the University Hospitals Birmingham NHS Foundation Trusts store plentiful amounts of EHRs/EMRs data. It would be worth evaluating the feasibility of emulating trials using specifically these EHRs/EMRs data, especially given the recent advances in health informatics (e.g. NLP) that enable quick access to and full use of these data. If these trial emulations are proven to be feasible and do indeed provide valid findings, these approaches could then be applied on a wider scale in order to gain scientific insights at a fast pace and with lower cost.

## Conclusions

This study reviewed explicit attempts of trial emulation studies across all medical fields and provides a comprehensive overview of the types of observational data that were leveraged, and the statistical methods used to address the following biases: (A) confounding bias, (B) immortal time bias and (C) selection bias. Different methods can used to address those biases. Future trial emulation studies should clearly define the causal question of interest, specify the protocol of the ‘target trial’, explain how observational data were used to explicitly emulate the ‘target trial’ and include this information in the paper. By doing so, reporting of trial emulation studies will be improved. When working with observational data, and if possible, the ‘target trial’ framework should be used as it provides a structured conceptual approach to observational research.

Although EHR/EMRs databases have been used successfully for trial emulation purposes, these consist of EHRs/EMRs data extracted from multiple sources and tend to use structured data. Currently, it remains to be determined whether EHR/EMRs data from a single healthcare facility include sufficient information and if this information is accurate enough to successfully emulate trials. If that is the case, EHR/EMRs data could be leveraged to improve patient care at the facility.

### Supplementary Information

**Additional file 1.** Search strategy for Medline(Ovid) platform.

**Additional file 2.** Data. The file contains all the information extracted from the 38 reviewed studies.

## Data Availability

The authors confirm that the data supporting the findings of this study are available within the article and its supplementary materials.

## Abbreviations

BADBIR: British Association of Dermatologists Biologic and Immunomodulators Register
BHF: British Heart Foundation
BladderBaSe: The Bladder Cancer Data Base Sweden
CALIBER: ClinicAI research using Linked Bespoke studies and Electronic health Records
CER^2^: The Comparative Effectiveness Research through Collaborative Electronic Reporting Consortium
CKD: Chronic Kidney Disease
COHERE: The Collaboration of Observational HIV Epidemiological Research in Europe
COVID: Coronavirus disease
CPRD: Clinical Practice Research Datalink
EHRs: Electronic health records
EMRs: Electronic medical records
ESRD: End stage renal disease
GHS: Geisinger Health System
HES: Hospital Episode Statistics
HIV: Human immunodeficiency virus
HIV-CAUSAL: HIV Cohorts Analyzed Using Structural Approaches to Longitudinal data
HPFS: The Health Professionals Follow-up Study
ICU: Intensive care units
IPCW: Inverse probability of censoring weighting
IPTW: Inverse probability of treatment weighting
ITT: Intention-to-treat
KCH: King’s College Hospital
KCL: King’s College London
NA-ACCORD: The North American AIDS Cohort Collaboration on Research and Design
NCDB: National Cancer Database
NCRAS: The National Cancer Registration and Analysis Service
NDB: The National Database of Health Insurance Claims and Specific Health Check-ups of Japan
NHS: National Health Service
NIHR ARC: National Institute for Health Research Applied Research Collaboration
NLP: Natural language processing
NPR: National Patient Register
ONS: Office for National Statistics
OPTN: Organ Procurement and Transplant Network
PDR: Prescribed Drug Register
PP: Per-protocol
PRISMA: Preferred Reporting Items for Systematic Reviews and Meta-Analyses
RCTs: Randomised controlled trials
REIN: The French Renal Epidemiology and Information Network
ROI: Republic of Ireland
RRT: Renal replacement therapies
SHCS: The Swiss HIV Cohort Study
SNRUBC: The Swedish National Register of Urinary Bladder Cancer
SRTR: Scientific Registry of Transplant Recipients
STOP-COVID: The Study of the Treatment and Outcomes in Critically Ill Patients with COVID-19
THIN: The Health Improvement Network
TMLE: Targeted maximum likelihood estimation
UK: United Kingdom
US: United States
USRDS: The United States Renal Data System
VA: The Department of Veterans Affairs
VACS: The Veterans Aging Cohort Study
