# Supplementary material for: Implementation of the trial emulation approach in medical research: a scoping review
Source: BMC Med Res Methodol. 2023 Aug 16;23:186. doi: 10.1186/s12874-023-02000-9 (PMC10428565; doi:10.1186/s12874-023-02000-9)
Supplement: Supplementary file 1 — Additional file 1. Search strategy for Medline(Ovid) platform. [file 12874_2023_2000_MOESM1_ESM.docx]

**Additional file 1: Search strategy for Medline(Ovid) platform**

Description: The file includes the search terms and the search strategy used to identify trial emulation studies in Medline(Ovid). The same search terms and search strategy were used in the other bibliographic databases. (DOCX 16kb)

1. trial emulation*.mp.

2. target trial*.mp.

3. pseudotrial*.mp.

4. emulated trial*.mp.

5. 1 or 2 or 3 or 4

6. exp observational study/ or observational.mp.

7. big data.mp. or exp big data/

8. exp case control study/ or case-control.mp.

9. exp cohort analysis/ or cohort.mp.

10. exp electronic medical record/ or electronic medical record*.mp.

11. electronic health record*.mp. or exp electronic health record/

12. routinely-collected data.mp.

13. real-world.mp.

14. exp data base/ or healthcare database*.mp. or exp health care/

15. exp longitudinal study/ or longitudinal.mp.

16. exp register/ or register*.mp.

17. exp retrospective study/ or retrospective.mp.

18. exp prospective study/ or prospective.mp.

19. registr*.mp.

20. 6 or 7 or 8 or 9 or 10 or 11 or 12 or 13 or 14 or 15 or 16 or 17 or 18 or 19

21. 5 and 20
